# Fast and Accurate Fitting and Filtering of Noisy Exponentials in Legendre Space

**DOI:** 10.1371/journal.pone.0090500

**Published:** 2014-03-06

**Authors:** Guobin Bao, Detlev Schild

**Affiliations:** 1 Department of Neurophysiology and Cellular Biophysics, University of Göttingen, Göttingen, Germany; 2 DFG Cluster of Excellence 171, Göttingen, Germany; University of California, Irvine, United States of America

## Abstract

The parameters of experimentally obtained exponentials are usually found by least-squares fitting methods. Essentially, this is done by minimizing the mean squares sum of the differences between the data, most often a function of time, and a parameter-defined model function. Here we delineate a novel method where the noisy data are represented and analyzed in the space of Legendre polynomials. This is advantageous in several respects. First, parameter retrieval in the Legendre domain is typically two orders of magnitude faster than direct fitting in the time domain. Second, data fitting in a low-dimensional Legendre space yields estimates for amplitudes and time constants which are, on the average, more precise compared to least-squares-fitting with equal weights in the time domain. Third, the Legendre analysis of two exponentials gives satisfactory estimates in parameter ranges where least-squares-fitting in the time domain typically fails. Finally, filtering exponentials in the domain of Legendre polynomials leads to marked noise removal without the phase shift characteristic for conventional lowpass filters.

## Introduction

Processes with linear first order kinetics, i.e. 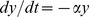, are ubiquitous in all fields of science, above all in the life sciences, chemistry, physics and engineering. Noisy exponentials are therefore among the most common experimental outcomes. Typically, noise removal is done by conventional lowpass filtering, and the function's parameters, ie, amplitudes and time constants, are retrieved by nonlinear least squares fitting (NLLSQ) such as the Levenberg - Marquardt algorithm (LMA) [1]–[3]. However, lowpass filters in the Fourier domain distort the signals by introducing phase shifts, and the LMA is too time-consuming in cases where the parameters need to be obtained rapidly or, equivalently, where exponentials need to be fitted in parallel. We therefore set out to find a faster method of filtering and fitting exponentials with the same or higher precision. The goal was to transform noisy signals such as exponentials into a space where signal and noise are mapped essentially onto different subspaces. It turned out that the method described herein is not only faster but in many practical applications also more precise than the LMA. In addition, it allows effective noise removal.

## Results

### Legendre filter

**Figure 1:**
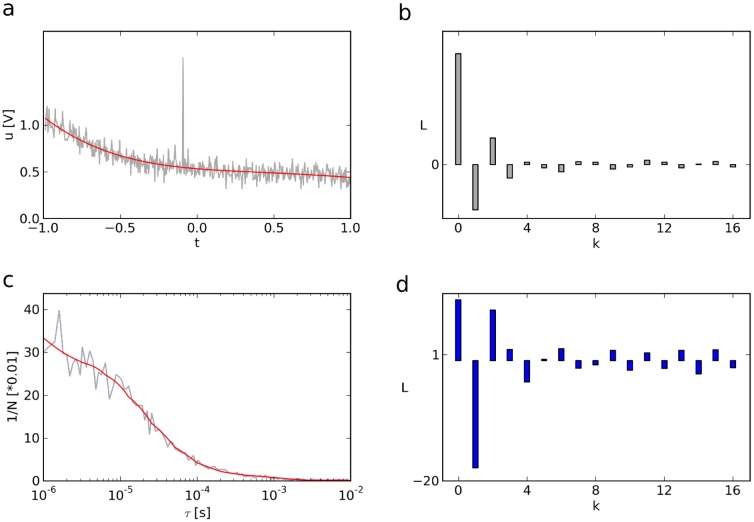
Filtering exponentials and Legendre lowpass. (**a**) Double exponential decay 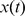 during a stopped-flow recording. The reaction monitored is the interaction of ruthenium complexes with DNA, scaled to the interval [−1, 1]. For the experimental details of the system, see [12]. (**b**) The first 17 components of the Legendre spectrum of 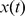. The inverse fLT of the components 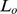 through 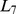 of the spectrum (**b**) gives the red curve in (**a**). Note that the sharp peak in the noisy trace is virtually not reflected in the filtered curve. (**c**) Autocorrelation curve (gray) resulting from an experiment where the diffusion constant of tetramethylrhodamine was measured (own data). In this example, fLT and ifLT are performed for non-equidistant samples, and we re-scaled the x-axis to correlation delays. (**d**) Legendre spectrum of the ACF shown in (**c**). The red curve in (**c**) is the inverse fLT of the components 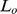 through 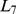 of the Legendre spectrum.

Consider the outcome of the stopped-flow experiment shown in **Fig. 1a** as well as its Legendre spectrum (**b**), obtained by the finite Legendre transform (fLT, see Methods). As Legendre polynomials are orthogonal on the interval [−1, 1] (**Fig. S1**), the first and the last sample of the time function are assumed to occur at -1 and 1, respectively (for scaling and re-scaling of time, time constants and amplitudes, see Methods). The Legendre spectrum (**b**) is plotted for the first 17 components, each of which indicates the contribution of a specific Legendre polynomial to the signal (eq. 4). While the inverse finite Legendre transform (ifLT) of the entire spectrum would, of course, reconstruct the original noisy function (**a**), the ifLT of the first, e.g. eight, components of the spectrum results in considerable noise removal (**a**, red). In analogy to Fourier theory, and for the sake of brevity, we name this filter Legendre lowpass. Its effectiveness is due to the fact that smooth signals such as exponentials are mainly represented by lower Legendre polynomials, i.e., by lower order components of the Legendre spectrum, while the signals' noise is mapped predominantly to higher order components (**Fig. 1** and **Fig. S2**).

**Figure 2:**
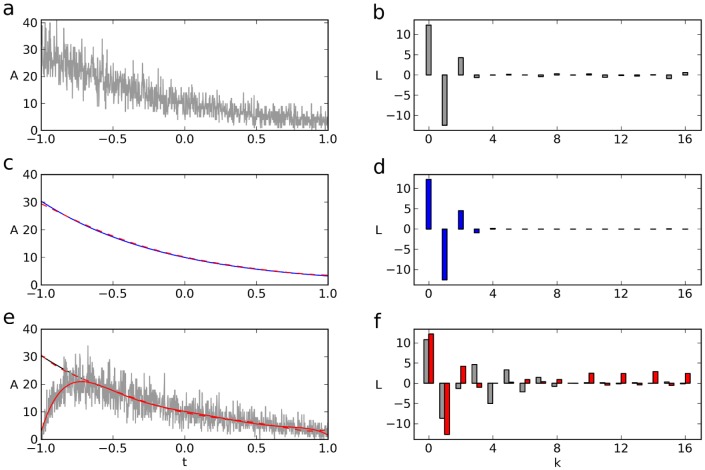
Filtering exponentials convolved with a system response function. (**a**) Exponential on the interval [−1, 1] with Poisson noise added. Amplitude, 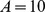, time constant 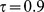. (**b**) Legendre spectrum of x as resulting from eq. 5. (**c**) Mean 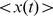 of 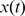 (continuous) and inverse fLT (dashed, eq. 6) of 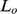 through 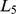 of the spectrum shown in **b**. (**d**) Legendre spectrum of the mean 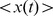, largely lacking higher noise components. (**e**) Noisy curve is the convolution of 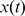 with 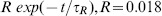 and 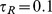. 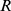 was chosen such that the curve overlaps with 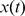 for large 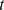. (**f**) Legendre spectrum (gray bars) of convoluted noisy exponential shown in **e** (continuous curve). The lowpass-filtered inverse transform is shown in **e** (continuous curve) and approximates the convoluted noisy exponential. In addition, **f** shows the Legendre spectrum of 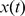, obtained through eq. 9. The lowpass-filtered inverse transform of this spectrum is shown as the red dashed curve in **e** and approximates the original non-convoluted exponential, from which the noisy convoluted curve was generated.

In practical cases, the output of a linear experimental device is affected by the system's response function, 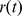. Often the response function is a lowpass so that 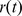 is an exponential itself. This can be neglected if its time constant 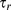 is much smaller than the 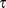 under investigation (as in the case of **Fig. 1a**). Generally, however, an experimental outcome 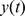 is the convolution of the signal, 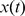 (**Fig. 2a**), with the response function, i.e., 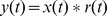. **Fig. 2e and f** show that the convoluted exponential 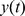 (**e**, noisy trace) can be approximated by Legendre filtering (**f**, gray, and **e**, red, cont.). Moreover, in many cases one is interested in the parameters of the original, non-convoluted function 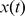 rather than in 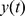. Obtaining these in the time domain (t-domain) from 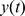 would require a deconvolution, which is notoriously inaccurate for noisy functions, and often not feasible. In contrast, an approximation of 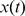 can readily be obtained in the Legendre domain (L-domain). To this end we calculate the Legendre spectrum of 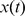 from 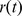 and the Legendre spectrum of 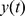 using eq. 8. The inverse transform of its lowpass-filtered spectrum (**f**, red bars) gives an approximation of 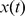 (**e**, red, dashed; eq. 5).

**Figure 3:**
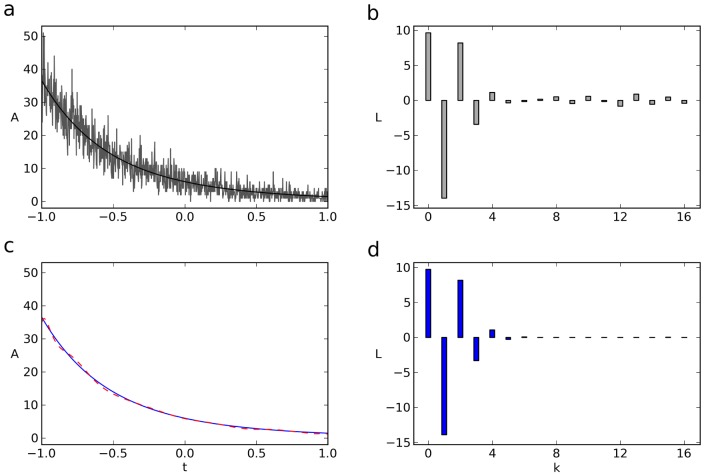
Legendre filter of the sum of two exponentials. (**a**) Noisy double exponential and its mean (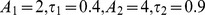). (**b**, **d**) Legendre spectra of the noisy double exponential (**b**) and its mean (**d**). (**c**) Mean of **a** (cont.) and lowpass-filtered Legendre spectrum in **b** (red, dashed).

**Figure 4:**
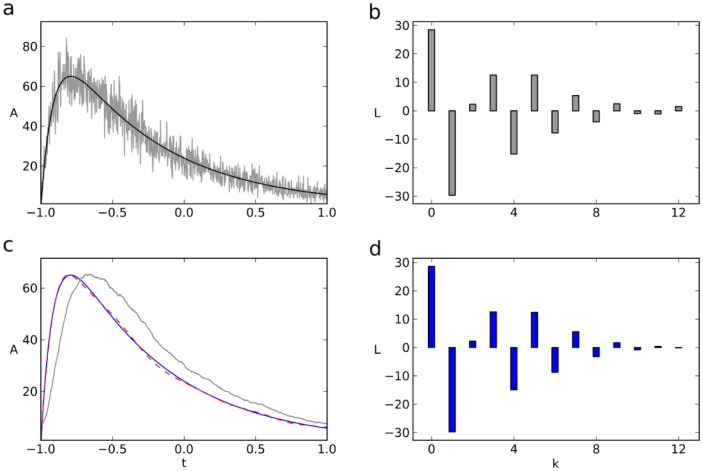
Legendre lowpass-filtered EPSP. (**a**) Noisy EPSP and its mean simulated as 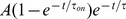, with 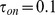, 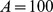 and 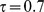. (**b, d**) Legendre spectra of the noisy EPSP (**b**), and its mean (**d**). (**c**) Inverse transform of 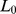 through 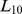 of **b** approximating the EPSP's mean (dashed, red). Gray curve in **c**, Fourier lowpass (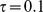) of the noisy EPSP.

The marked reduction of noise observed could also be observed when a sum (**Fig. 3**) or a product (**Fig. 4**) of noisy exponentials was analysed. The product of exponentials could serve as a model, e.g., for excitatory postsynaptic potentials (EPSPs) of neurons. Here it is interesting to compare the Legendre lowpass-filtered EPSP (**Fig. 4c**, dashed, red, calculated from the lower components of the EPSP's Legendre spectrum, **b**) with the conventionally lowpass-filtered EPSP, which exhibits the characteristic phase shift of Fourier lowpass-filters (**c**, gray). For obtaining parameters such as time-to-peak and time constants, the Legendre lowpass clearly appears to be more appropriate than the Fourier lowpass.

The noise removal effect of Legendre filters is by no means limited to the above functions as shown for the autocorrelation function (ACF) of an fluorescence correlation spectroscopy (FCS) experiment (**Fig. 1c**). Its Legendre spectrum as well as the Legendre lowpass-filtered ACF are shown in **d** and **c** (red), respectively. Though this cannot replace the fitting of FCS data, it allows a filtered online representation of the ACF.

Taken together, the finite Legendre transform compresses the data from the number of samples in the t-domain to a small number of Legendre components. Second, considerable noise reduction can be achieved by Legendre lowpass-filtering, and third, deconvolution of noisy exponentials can conveniently be carried out in Legendre space.

At this point the question arises whether the parameters 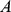 and 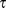 of noisy exponentials can be retrieved in the L-domain, and whether this is faster and as least as precise as fitting in the t-domain. Our strategy to analyze these questions is illustrated in **Fig. S2**. A noisy exponential, which might look similar to part g of the figure, is first transformed (fLT) to its Legendre spectrum. The lower components of the Legendre spectrum are then fitted to the corresponding, amplitude- and time constant-dependent components of a pure exponential (eq. 4). For the comparison of this method with the LMA in the t-domain, we simulated noisy exponentials (g). First, we defined the amplitude and time constant of an exponential (a) and then we added Poisson noise (c) as well as an offset with gaussian noise (e) to it. The right column of the figure clearly shows that the Legendre spectrum of (a) has only low components, while the transforms of (c) and (e) are characterized predominantly by high components. The LMA fit of the resulting noisy exponential in the t-domain (g) now gives estimates 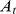 and 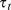 for the true amplitude 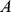 and true time constant 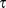, while the LMA fit of its lower Legendre components (h) gives estimates 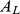 and 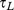 for 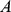 and 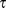 in the L-domain.

### Accuracy and precision of 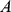 and 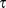 retrieval: t-domain versus L-domain

**Figure 5:**
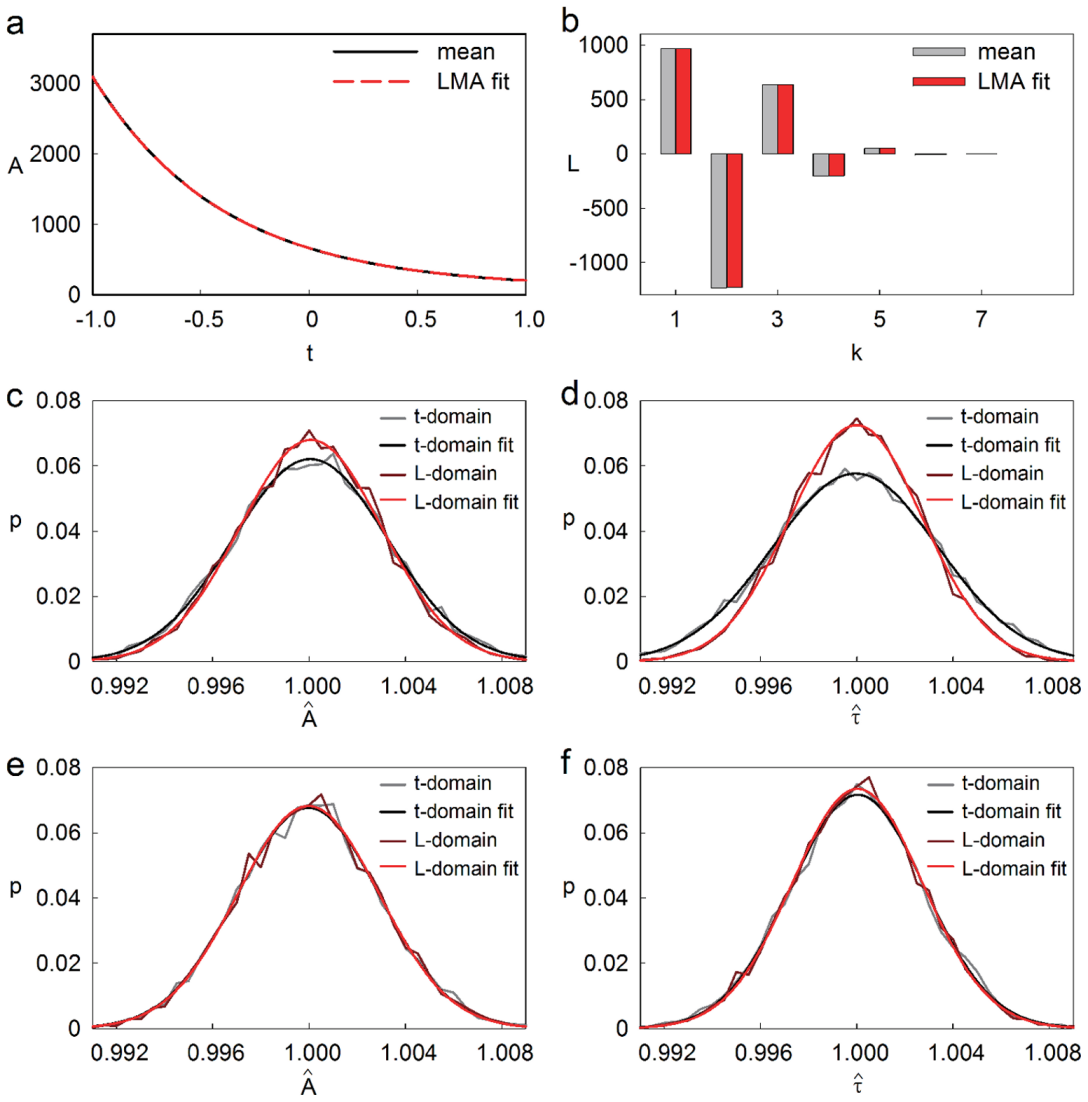
Comparison of fitting in time- and Legendre - domain. (**a**) LMA fit (dashed, red) and mean (cont.) of a noisy exponential with offset in the time domain. Poisson noise; offset, 100; gaussian offset noise with 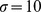. (**b**) Legendre spectrum of the same noisy exponential (gray) and LMA fit in the Legendre space (red). (**c,d**) Probability density function giving the frequency with which the fitted amplitude, normalized to the true value (**c**), or with which the fitted time constant, normalized to the true value (**d**)occurs in 5000 trials. True values assumed in c,d: A = 3000, tau = 0.1. All weights set to 1. The red and gray curves give the frequencies resulting from the fits carried out in the time (gray) or Legendre (red) domain, respectively. (**e,f**) Probability density functions as in (**c,d**), except that the data were weighted with 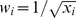. The four calculated pdf's in **c** through **f** are fitted by Gaussians.

To assess and compare the precision and accuracy of fitting in the t-domain versus the L-domain, we used simulated data as described and applied the LMA with equal weights in both domains, thereby obtaining 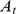 and 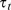 as well as 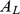 and 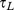. Either way of fitting can be done with the pure signal 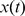 or with 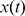 being convolved with a system response function 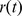. **Fig. 5a** shows the exponential with the true paramters 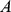 and 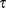 together with the LMA fit of its noisy variant in the t-domain, yielding 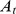 and 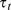. On the other hand, **Fig. 5b** shows the Legendre spectrum of the noisy exponential (gray bars) along with the LMA fit in the L-domain (*adjacent red bars*), yielding 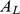 and 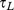. In this example we obtain 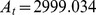 and 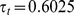 on the one hand and 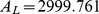 and 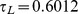 on the other. Both pairs approximate the real values 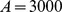 and 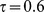, whereby, in this case, 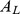 and 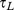 come closer to 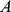 and 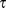. Another realization of the same experiment (same 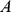 and 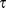) would, of course, lead to a different pair of ‘best fits’ and the LMA in the t-domain might come closer to 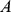 and 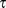 this time. We therefore analyzed a large number of realizations (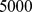) of the same experiment, fitted them using either method, and plotted the distributions of the normalized fitted values 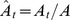 and 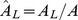 (**Fig. 5c**) as well as 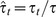 and 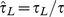 (**Fig. 5d**). Both amplitudes and time constants are estimated with higher precision in the L-domain, the effect being more pronounced for the time constants. The precision of fitting 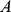 and 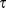 in the L-domain or the t-domain can be quantified by the Gaussian sum of variances, resulting in the joint errors 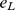 and 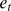 (see Methods, eq. 14a,b). The probability for the error in the L-domain, 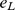, to be smaller than 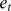 varied as a function of 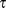 and was found to lie in the range between 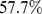 and 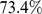.

In the above comparison, we have used the LMA with equal weights, 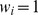, for all samples. Thereby we have implicitly assumed a type of measurement, where no a-priori knowledge is available concerning the measurement and its noise. In such cases and in particular when it is unclear whether the data contain one or two exponentials, and one of them might be suppressed by the importance weighting, all weights should be set to 1. Only in cases where reasonable assumptions on measurement errors can be made, which is, of course, the case for our simulated data, an appropriate weighting should be chosen, since this improves the estimation of the parameters. In fact, the comparison of precision and accuracy of the same data as above but with optimal weighting for Poisson noise, shows virtually no difference between the fitting in L- and t-domain (**Fig. 5d and e**). However, gradually increasing the gaussian noise renders this way of importance weighting rapidly sub-optimal (**Table 1**), and the fit in the L-domain gives the accurate parameters more frequently. Fitting in the L-domain thus appears to be equivalent to optimal weighting in the t-domain. This is useful because the optimal weights in the t-domain are mostly not known.

**Table 1:** Comparison of fitting in L-domain and t-domain of simulated noisy exponentials defined by parameters 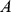 and 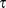, superimposed Poisson noise and stationary gaussian noise with different standard deviations 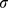.

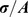	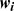	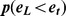
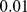	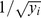	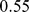
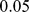	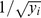	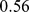
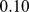	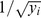	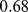
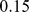	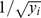	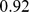

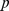
 is the probability that the fitting parameters obtained in the L-domain approximate the true values better than those obtained in the t-domain (i.e., 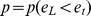) under the indicated noise condition. With optimal importance-weighting for Poisson noise (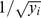), the comparison is carried out for four different levels of gaussian noise, given as a 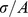 (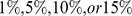).

### Optimal number of Legendre components

**Figure 6:**
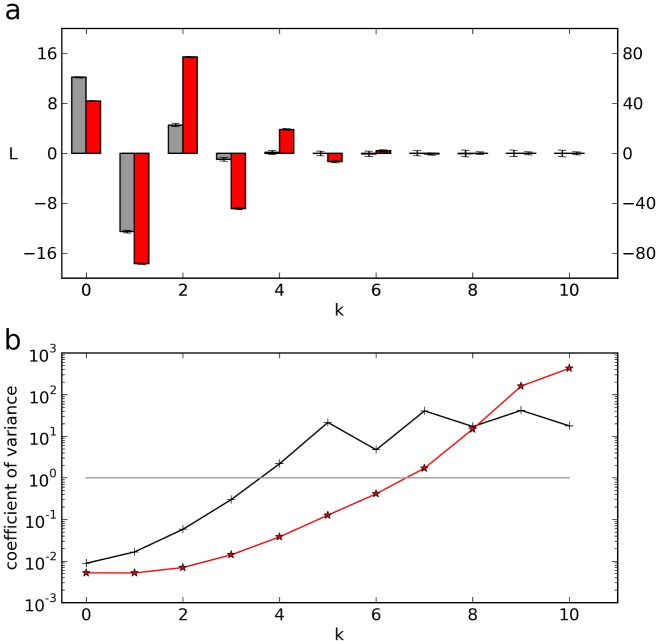
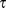
- dependence of the required number of Legendre components. (**a**) Legendre spectra of noisy exponentials with 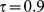 (gray) and 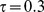 (red). Shown are the average Legendre amplitudes obtained from 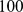 trials. Error bars, standard deviation of the respective component. (**b**) Coefficient of variation of the components for the two spectra shown in **a**.

In the above, we used the first eight components of a Legendre spectrum for calculating the inverse transform (**Fig. 1a,b**) and for the comparison of accuracies in the t- and L-domain (**Fig. 5c,d**). However, the spectral amplitudes' decays in the latter figure suggest that five or six components might have been sufficient to obtain the same result. This and similar observations led us to analyze the question of how many Legendre components should be taken into account for the LMA in the L-domain. The answer turned out to depend on the ratio of 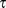 and the time 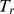 over which the record was taken. This can be seen qualitatively by comparing the Taylor series representation of exponentials with the Legendre representation, and quantitatively by calculating the coefficient of variation of Legendre components (**Fig. 6**). Specifically, the Taylor series representation of an exponential (eq. 9) shows that the terms of the Taylor series decay with 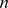 as 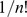, but that small time constants 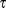 counteract this decay, since (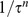) increases with decreasing 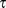. As a consequence, for the same accuracy smaller 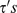 require higher orders of the Taylor series. Clearly, as there is a unique mapping from the Taylor series representation to the Legendre polynomial representation of exponentials (eq. 12), the number of Legendre components required increases for decreasing time constants.

As a quantitative way of obtaining an appropriate cut-off index 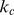 of Legendre components we used the coefficient of variation 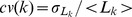, which describes, for each k, the ratio of the standard deviation of 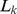 to its average, i.e., the ratio of noise to signal. For a noisy exponential with 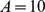 and 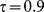, the Legendre components including their standard deviations are shown in **Fig. 6a** (gray). Up to the forth component, the standard deviations are rather small and can hardly be recognized. The corresponding 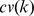 is plotted in part **b** of the figure (black). Assuming 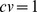 as threshold for an acceptable signal-to-noise ratio, we obtain 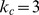 as cut-off. In the case of a shorter time constant 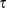, e.g. 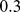, the standard deviations can virtually not be recognized in the spectrum (**Fig. 6a**, red) but the 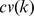 clearly show that up to 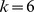 the signal dominates the noise. It can be taken as a rule of thumb, that for 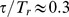 six Legendre components give optimal results.

### Computational cost

**Figure 7:**
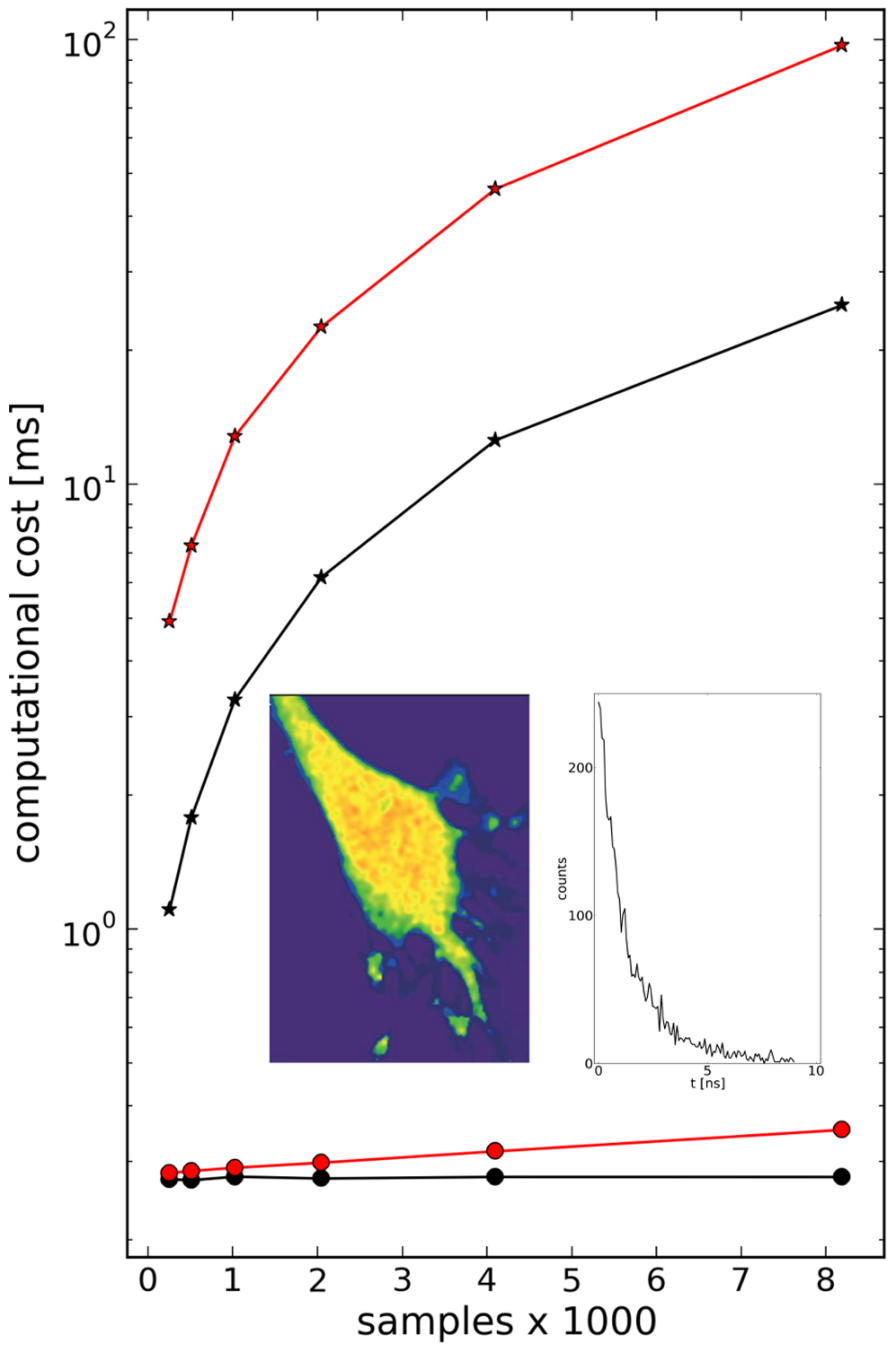
Computational cost of fitting in time - and Legendre - domain. Dots and asterisks indicate the computational cost in Legendre - and time - domain, respectively, on a logarithmic scale. Black and red curves refer to whether (red) or not (black) a system response function was taken into account. Each point or asterisk is the average duration of 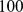 computations. As the analysis in the time - domain is fastest when using the Fast Fourier Transform, we choose the sample sizes to be powers of 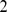, starting with 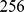. Inset, left, FLIM image of a mouse hippocampus neuron probed for oxidative stress using the hydrogen peroxide sensor HyPer [13] and, right, fluorescence lifetime function for one pixel in the middle of the cell (150 samples, data courtesy K. Kizina and M. Müller, CNMPB, Göttingen).

For comparing the computational cost in the t-domain versus the L-domain, we started out with FLIM data from an experiment, where oxidative stress was measured in cultured hippocampal neurons using the HyPer sensor and two-photon microscopy (**Fig. 7**). For the 150 data samples recorded the computational cost was 8.78 ms/px (t-domain) versus 0.3 ms/px, amounting to approx. 575 s (t-domain) versus 19,7 s (L-domain) for the whole image. Given an image acquisition time of 30 s, the analysis of exponentials in the L-domain has become feasible in real-time.

As the algorithm does not depend on the specific type of experiment, this result can readily be generalized to higher sample sizes. Expectedly, the computational cost of the LMA in the t-domain is approximately proportional to the number of samples. On the other hand, fitting in the L-domain requires not only (i) the fitting in the L-domain but also (ii) the calculation of the finite Legendre transform (fLT), and the rescaling of the parameters (see **Methods**). The first step is much more time-consuming than (ii) and (iii), but it is independent of the number of samples in the t-domain. We analyzed this quantitatively and carried out both fitting procedures for increasing numbers of experimental samples.

Under our conditions (Intel i7, 2.3 GHz, Python/Numpy), fitting in the L-domain took less than 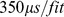 and was indeed independent of sample size (**Fig. 7**, black dots), whereas fitting in the t-domain was approximately 12 to 100 times slower for 1024 to 8192 samples, respectively (**Fig. 7**, black asterisks). In case the recorded function 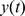 is a convolution, 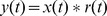, fitting in the t-domain takes approximately four times longer (**Fig. 7**, red asterisks). This is because a convolution of the model function with 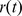 has to be carried out for each minimization step, as the direct deconvolution of 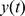 is often not practicable due to the superimposed noise.

On the other hand, fitting the Legendre spectrum of 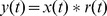 is almost as efficient as fitting 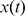, since this requires only one additional step, namely a matrix multiplication (eq. 8). As this operation involves the samples size, the computational cost increases accordingly, though rather moderately (**Fig. 7**, red dots). The same applies to the computation of the fLT itself, which took from 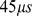 (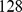 samples) to 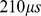 (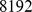 samples) for seven components.

Finally, the computational cost depends also on the initial parameter values used. It turned out that calculating the Legendre spectrum of the experimental outcome and solving the first three lines of the system eq. 12 gives a first guess of the three parameters, 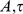, and offset, which can be used as starting values, also for the fitting of double exponentials.

### Double exponential decay

**Figure 8:**
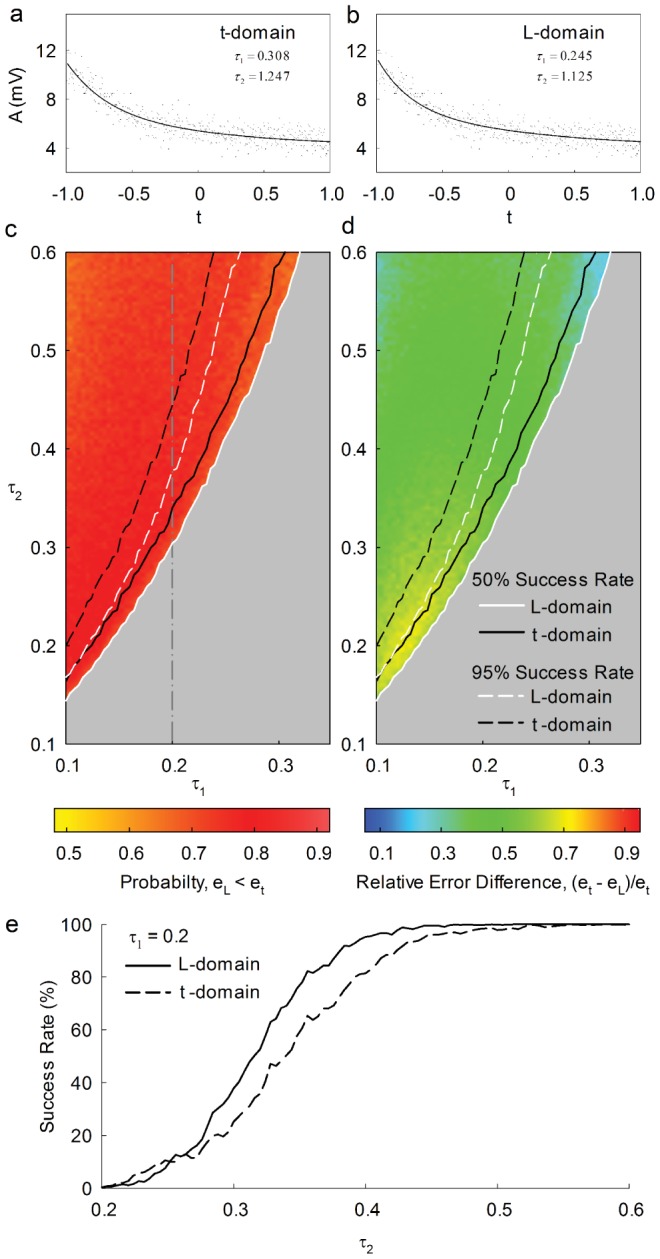
Double exponential decay analysis in time- and Legendre-domain. (**a,b**) The interaction of Ru(II) complexes with DNA (same as in in Fig. 1) shows double exponential decay([12], data courtesy of F. Secco, Univ Pisa,I). Same data fitted in t- (**a**) and L-domain (**b**). (**c**) Probability for the fitting error in the L-domain, 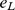, to be smaller than that in the t-domain, 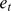, represented as a function of 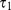 and 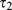. Color code of the probability is shown beneath the plot. For each pair (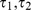) 1000 trials were computed. (**d**) Relative difference of fitting errors, 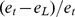, as a function of 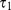 and 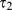. Color code beneath the plot. 1000 trials per pixel. Left of the solid and dashed white lines in **a** and **b**, the success rate of the fit in the L-domain is larger that 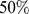 and 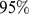, respectively. Left of the solid and dashed black lines in **c** and **d**, the success rate of the fit in the t-domain is larger that 50% and 95%, respectively. Relative error differences were calculated only for successful trials. (**e**) Success rate of fitting in the L-domain (solid) and t-domain (dashed) along the vertical line in **c**, i.e., as a function of 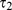 with 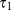 kept at 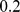.

The analysis of double exponential decays is relevant in many instances but unfortunatety, at least in many biological recordings, the underlying molecular processes are non-stationary so that the experiment cannot be repeated under *identical* conditions. A typical situation is a decay curve from experimental data which at first glance seems to be a double exponential and which needs to be analyzed. However, when analyzed in t-domain and L-domain, the same data usually give different results for the parameters as shown in **Fig. 8a and b**. It was therefore indispensable to analyze which of the two methods is superior in the sense that the resulting parameters come closer to the true values. We therefore generated, on the basis of two known amplitudes and time constants, a large number (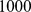) of double exponentials, added Poisson and gaussian noise as above, and fitted them in both the t- and L-domain using the LMA in either domain. In addition to having the resulting 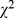 values as a measure to judge the quality of fits, we also used the individual errors 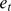 or 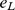 as a measure of how much the individual fitted parameter values deviated from the (known) true ones at minimized 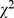. This way, the fitting result can be observed separately for amplitudes and time constants.

As four parameters 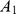, 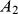, 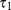 and 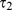 have to be taken into account now (rather than two as above), the corresponding terms for 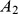 and 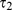 need to be added to eq. 14a and b, respectively. **Fig. 8c** shows for the relevant range of 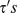 that the double exponential fit in L-domain is more likely to give a better approximation than the corresponding fit in t-domain, and **Fig. 8d** shows how much the error in the t-domain differs from that in the L-domain. We calculated, for either domain, the success rate 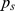 of the fit. 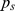 was obtained as the frequency, with which the algorithm results in two different amplitudes and time constants, while the correponding failure rate, 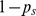, describes the cases, in which the algorithm converges to only one time constant or where it does not converge at all. The success rate 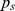 for 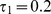 (dashed vertical line in **Fig. 8c**) is plotted in **e**. Clearly, for 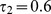, both methods converge to double exponentials in 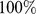 of all trials. With decreasing 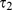, however, the success rate decreases too, the decrease being more pronounced in the t-domain (**e**, dashed). In **Fig. 8c,d** we have marked the 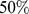 and the 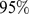 - thresholds of the success rate by continuous or dashed lines, respectively. In these regions, the probability 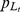 of more accurate results in the L-domain varied between 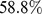 and 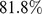 (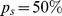) and between 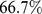 and 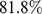 (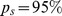). Taken together, for the parameter region where both ways of analysis (i.e., t-domain and L-domain) are successful, the fitting results in the L-domain come, on the average, closer to the true double exponential decay parameters. Furthermore, the minimum ratio of 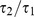 that can be differentiated is smaller for the fit in the L-domain (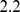 vs. 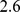).

## Discussion

The method proposed herein consists of two steps, first the deterministic transform of the signal into the L-domain, and second, NLLSQ/LMA fitting of the lower Legendre components. The major advantage of the method is the gain in analysis speed, which is primarily brought about by the fact that the LMA is applied to 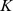 Legendre components rather than to the 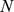 samples in the t-domain, with 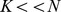. At the same time, the method yields the accurate parameters with high precision, which is essentially brought about by largely separating signal and noise in the L-domain with subsequent parameter estimation from a truncated Legendre spectrum.

The situation may be compared to a spectrally narrow-banded signal buried in noise, e.g., a very faint radar or radio carrier frequency, where the carrier can be detected in Fourier space or by cross-correlation, because the noise power is small that falls into the signal's spectral range. Likewise, exponentials can be well detected in the L-domain, because the noise power that falls into the lower components, where the signal is mapped, is low.

Legendre polynomials are important functions in many areas of physics [4]. They are orthogonal on the interval [−1,1] which allows expansion of any function that is continuous on this interval into its spectral Legendre components. The operation by which the spectrum is obtained has been called Legendre transform (e.g. [5], [6]). Though this usage of the term appears straightforward, it is ambiguous, since in the canonical language of physics and chemistry, Legendre transformations are well-known and widely used to express a function 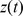 in terms of its derivative rather than in terms of its independent variable 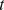 [7]. We therefore follow Jerri [4] and Méndez-Pérez and Morales [6], and name the operation by which the Legendre spectrum is obtained (eq. 4) *finite Legendre transform (fLT)*, in close analogy to the finite Fourier transform, which is also carried out over a finite interval.

The Legendre spectra of a noisy exponential and its noise-free variant differ in the amplitudes of their components. While the higher components vary considerably with the noise in the time function, the lower components contain little noise, so that backtransforming them into the t-domain results in effective noise removal. In analogy to conventional lowpass filtering in the Fourier domain [8], this filter should be called Legendre lowpass. Evidently, not only exponentials can be lowpass filtered this way. We have, for instance, successfully checked this for the product of two exponentials (**Fig. 4**), for bleaching time courses in LSM imaging (not shown), and for fluorescence correlation spectroscopy data, where, in the simplest case, the autocorrelation function takes on the form 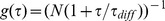, 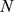 and 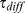 being constants (**Fig. 1c**). However, as this paper is concerned with exponentials, we did not investigate which other classes of functions can advantageously be Legendre lowpass filtered.

Apart from Legendre filtering, the lower Legendre components can also be used to obtain the exponentials' parameters by finding the Legendre spectrum that fits best, in the least squares sense, the fLT of the data. In practice this means that the LMA is applied to the lower Legendre components of an experimental record (or our simulation) using the correspondigly truncated and parameter-dependent Legendre spectrum of a noiseless exponential as the model. Prior to the actual fitting, an appropriate record length 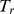 as well as a good guess of the 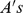 and 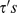 involved must be determined. The cases 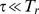 and 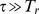 can be excluded for analysis, because in either regime the samples considered carry too little infomation about the exponential. Satisfactory fitting results are obtained in the range 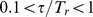, and the ratio 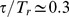 turned out to be the best choice. In a next step, we find a good guess for the initial values 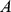 and 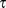 by comparing the Legendre representation of an exponential with its Taylor series. Both representations involve the powers of time, 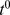, 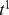, …, and the comparison of the respective coefficients leads to a system of linear equations, which allows calculating 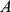 and 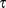 from the Legendre spectrum of the signal and the constant coefficients of the matrix P of Legendre polynomials. Due to the noise in the signal's Legendre components and the finite size of the system (eq. 12), these values for 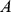 and 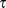 are, of course, mere approximations, but as such they are optimal starting values for the fitting procedure. (Obviously, the same initial values 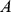 and 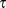 could also be used for least squares fitting in the t-domain.)

Fitting in the L-domain is much faster than in the t-domain, particularly if the exponentials under investigation are convolved with a system response function (**Fig. 7**). The computaional costs in time and L-domain correspond, in a good approximation, to 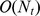 and 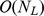, with 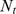 and 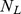 being the number of values to be fitted, respectively. This is highly relevant for time-sensitive applications such as fluorescence lifetime imaging (FLIM) or in patch clamp experiments.

In addition to being faster, the fits in the L-domain have the same or a higher probability of giving the accurate paramters 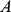 and 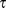. Without importance weighting of the data, fitting in the L-domain is always better (**Fig. 5**). Accordingly, fitting in the the L-domain is also superior to earlier algorithms which either do not converge as well as the LMA or are less precise than the LMA [9].

Regarding the comparisons of precision, a note of caution is necessary. Whenever assumptions on measurement errors can be made, an appropriate weighting should be chosen, since this improves the estimation of the parameters. However, in cases with little or no a-priori knowledge on measurement errors, optimal weights cannot be found, and fitting in the L-domain is more precise.

When analyzing double exponential decays, the probability for obtaining better parameter approximations is always higher in the L-domain. In addition, there is a range of the parameter space where fitting in the L-domain still gives satisfactory estimates, while the analysis in the t-domain fails to converge.

At the beginning of our study we also considered alternative filtering methods such the wavelet transform, Chebychev or Laguerre polynomials. However, the wavelet transform is slower than the fLT and also more appropriate for a different kind of signal, being defined in a given time and frequency window, while the Chebychev and (associated) Laguerre polynomials, albeit similar to the Legendre polynomials, are only orthogonal, when a weighting function applying to the inner product, which makes their use computationally less efficient.

Experimental outcomes often show an offset, which has to be taken into account when using the LMA in the t-domain. In Legendre space, the offset is simply added to the first component, i.e., the first component of the Legendre fit gives the constant amplitude plus the offset. A good guess for the offset can be calculated from eq. 10, where three equations, which have to include the first one, need to be solved to obtain estimates for the offset as well as for 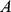 and 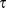 (the first equation then results in A plus the offset).

Historically, Legendre appears to play a double role in exponential fitting, since, apart from the fast parameter retrieval described herein, he was the first to publish the idea of least squares fitting in 1806 (though this method is mostly attributed to Gauss, who claimed the first usage of it) [10].

## Materials and Methods

### Simulated data and rationale of their usage

To simulate a set of noisy exponentials having the same amplitude and time constant, we first defined the amplitude 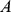 and the time constant 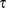 for this set. We then calculated the exponential 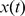 and added Poisson noise to it. Where indicated, we also added an offset and gaussian noise. We name the (noiseless) exponential 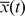 and the trials, which differ only in their noise, 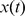. By definition, we thus have a-priori knowledge of 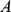, 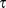, 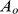, and 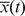. The sample size N was chosen to be 1000, except for the comparison of computational costs.

Simulated data and thus the a-priori knowledge of 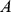 and 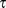 are necessary, since we need to compare the fitting results in both the t- and the L-domain with the real values of the parameters 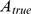 and 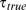. On the one hand, the direct application of the LMA to a noisy exponential 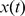 (ie, LMA in the t-domain) yields specific values 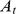 and 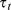, on the other, transforming the exponentials into the L-domain and applying the LMA to the lower components of the Legendre spectrum gives values 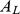 and 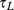. Thus, for every generated noisy curve we get four parameters 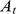, 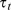, 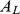 and 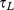. The ratios 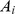/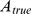, 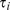/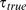, i meaning t or L, tell by which factor the fitted values deviate from the real parameters. A ratio of 1, for instance, indicates a perfect fitting result. Finally, plotting the probability density functions of the deviations reveals accuracy and precision of the fits in the t- and the L-domain (Fig. 2).

### Legendre polynomials and finite Legendre transform (fLT)

Legendre polynomials result from the orthogonalization of the powers 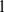, 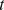, 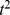, … leading to
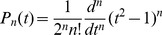
(1)with 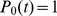. From this we have a recursive definition
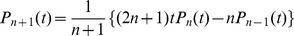
(2)with 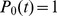 and 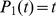. The first five Legendre polynomials are given by
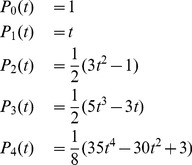
(3)and plotted in the **Fig. S1**.

Legendre polynomials are an orthogonal set of functions on the interval [−1,1], so that any function 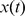 defined and continuous on this interval can be transformed into its Legendre spectrum by using the finite Legendre transform (fLT),
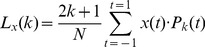
(4)


In our case, t is a discrete variable assuming N values between −1 and 1. The factor 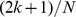 is a normalization factor.

The time function 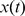 can be regained from its Legendre spectrum 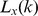 by the inverse fLT (ifLT),
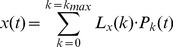
(5)


Alternatively, one might consider to use the shifted Legendre polynomials [11], which are defined as 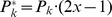 and orthogonal on the interval [0,1]. While this leads to the same results, it is computationally inconvenient, because the factor 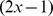 needs to be taken into account in a number of operations.

### Legendre spectrum of exponentials

Let us consider a class of stochastic processes 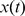 characterized by the parameters 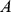 and 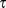 as well as by non-stationary means 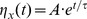, and Poisson-noise fluctuations.

We conveniently represent the respective experimental outcomes, i.e., the realizations 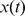 of such a process, in the space of Legendre polynomials 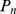. While the fLT 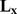 of 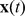 is a sequence of random variables 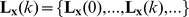, the fLT of any particular realization 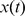 of 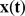 gives a specific Legendre spectrum, 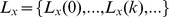, i.e., the coefficients of a linear combination of Legendre polynomials.

The first practical step of data analysis is to map the time inverval of the experimental results onto the interval [−1,1], i.e., we redefine the time axis so that the exponentials are spanned over [−1,1] rather than over 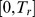, 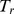 being the time of the last sample.

In a second step we calculate the components 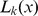 of the the Legendre spectrum (eq. 4).

In cases, where an experimental output 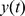 is the convolution of an exponential 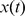 and a device response 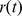, 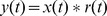, we can express 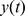 as
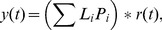
(6)whereby the 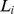 are the unknown Legendre spectrum components of the noisy exponential to be found. This equation can be rewritten in terms of a matrix 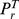 consisting of the transposed Legendre polynomials each convoluted with 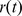


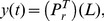
(7)with 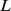 being the vector of (unknown) Legendre coefficients of the pure, non-convoluted exponential 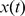 recorded. Using the pseudoinverse of 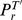, we obtain
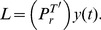
(8)


The pseudoinverse and the resulting Legendre components 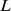 of the experimental outcome 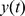 are calculated once only, prior to fitting (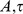) - dependent Legendre components to the experimental spectrum. With increasing sample size the calculation of L increases accordingly (**Fig. 7**).

From the inverse transform of the vector 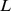 of Legendre coefficients, an approximation of the non-convoluted exponential can be obtained.

### Direct retrieval of amplitudes and time constants from Legendre components

The finding that the first spectral Legendre components are virtually identical for noisy and pure exponentials with the same parameters suggests a third and analytical way of obtaining 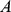 and 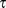. We start out with the Taylor series of an exponential,
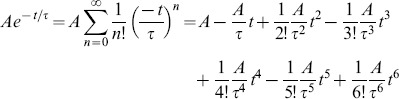
(9)and represent the same exponential by the superposition of Legendre polynomials
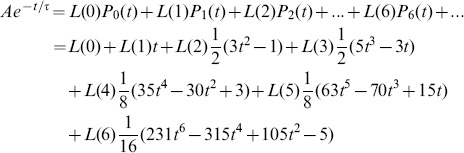
(10)


After rearranging with respect to the powers of t, i.e.,
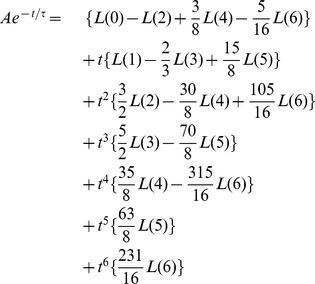
(11)a coefficient comparison with the Taylor series (9) gives 
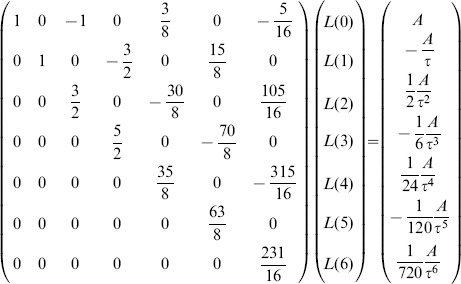
or
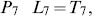
(12)where the index indicates the cut-off, 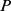 stands for the matrix of Legendre polynomial coefficients, 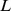 for the truncated Legendre component vector, and 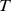 for the vector of Taylor series coefficients. In case the exponential tends to an offset value 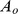, 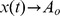 for 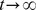, 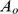 adds to the first term of the Taylor series and, consequently, to 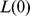


Taken together, the analytically derived equation 12 relates, for a given order, the Legendre components of an exponential to its paramters 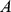 and 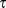. This is useful because it gives a good guess for 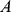 and 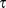 from the lower Legendre components.

### Fitting errors

As every fitted exponential 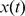 depends on 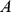 and 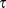, 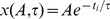, the resulting error of each value 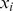 can easily be obtained from Gauss' error propagation, i.e.,
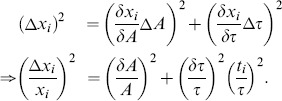
(13)


The total errors of a fit in the L- or the t-domain, 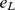 or 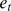, are thus
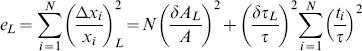
(14a)

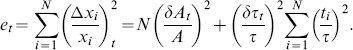
(14b)


### Scaling

If a function is to be fitted in Legendre space, it has to be scaled to the time interval [−1,1],
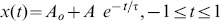
where 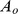 is an offset of the exponential. Prior to fitting this does not require any computation as we may assume the function's first and last value to occur at 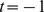 and at 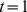 rather than at 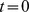 and 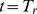. After fitting, the time scaling factor 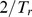 is needed to obtain the real time constants 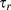. Likewise, the amplitude needs to be rescaled by the factor 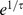, since, on the interval [−1,1], the real amplitude 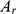 is the value at 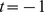, while the fit gives the amplitude value 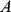 at the scaled time 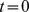. The exponential in the t-domain,
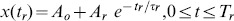
can thus be obtained by
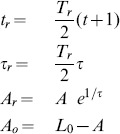
(15)


### Software

All calculations were carried out in Python/numpy/C. The code used in this paper, in particluar filtering routines, fitting routines, the Legendre polynomials, the fLT, ifLT, are available at the public “python package index” repository (https://pypi.python.og/pypi), and on our website (https://www.ukmn.gwdg.de/).

## Supporting Information

Figure S1 **Legendre polynomials.** Shown are the first five Legendre polynomials, their order being indicated at the respective curve.
(TIF)

Figure S2
**Signal and noise in time- and Legendre-domain.** (**a**) Exponential of known amplitude and time constant. **b**) Legendre spectrum of (a). **c**) Poisson noise corresponding to the amplitude of the exponential in (a) and **d**) its Legendre transform. **e**) Stationary gaussian noise and **f**) its Legendre transform. **g**) Noisy exponential, superposition of (a), (c), and (e), the latter showing gaussian noise with mean 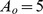 and standard deviation 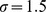. The mean simulates the offset of the measurent. **h**) Legendre transform of (g). For the comparison of methods, the LMA is applied in both domains, and the respective results are compared to the known parameters of the simulated data. 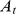, 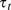, 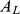, and 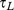 are estimates of the true values 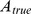, 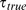, which, in the following, we call for short 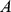 and 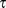.(EPS)
