# Correction: Fast and Accurate Fitting and Filtering of Noisy Exponentials in Legendre Space

**DOI:** 10.1371/journal.pone.0094711

**Published:** 2014-04-02

**Authors:** The PLOS ONE Staff

The citations in the fourth and fifth sentences of the third paragraph of the Discussion section were inadvertently switched. The correct sentences should read:

Though this usage of the term appears straightforward, it is ambiguous, since in the canonical language of physics and chemistry, Legendre transformations are well-known and widely used to express a function *z*(*t*) in terms of its derivative rather than in terms of its independent variable *t* [4]. We therefore follow Jerri [6] and Méndez-Pérez and Morales [7], and name the operation by which the Legendre spectrum is obtained (eq. 4) *finite Legendre transform (fLT)*, in close analogy to the finite Fourier transform, which is also carried out over finite interval.
