# Supplementary figures and images for: Fast and Accurate Fitting and Filtering of Noisy Exponentials in Legendre Space
Source: PLoS One. 2014 Mar 6;9(3):e90500. doi: 10.1371/journal.pone.0090500 (PMC3948392; doi:10.1371/journal.pone.0090500)

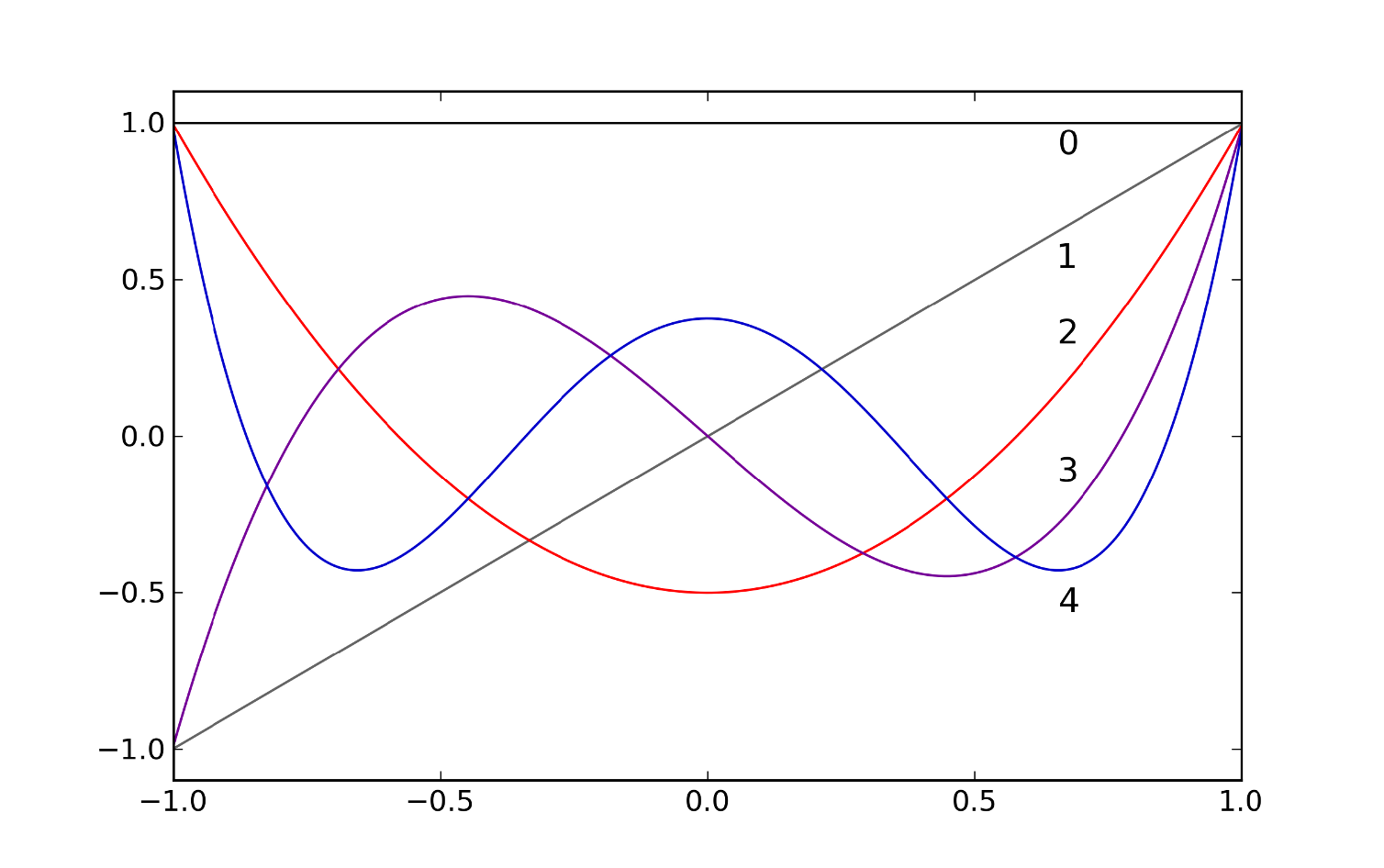

Supplement: Figure S1 — Legendre polynomials. Shown are the first five Legendre polynomials, their order being indicated at the respective curve. (TIF) [file pone.0090500.s001.tif]
